# Synthesis of novel Schiff bases containing arylpyrimidines as promising antibacterial agents

**DOI:** 10.1016/j.heliyon.2019.e02318

**Published:** 2019-08-20

**Authors:** Soukhyarani Gopal Nayak, Boja Poojary

**Affiliations:** Department of Chemistry, Mangalore University, Mangalagangothri, Mangaluru, 574199, Karnataka, India

**Keywords:** Materials chemistry, Natural product chemistry, Organic chemistry, Pharmaceutical chemistry, Pyridine, Schiff bases, Electron donating, Antimicrobial activity

## Abstract

Pursuing our recent interest regarding the antimicrobial activity of Schiff bases derivatives, we have synthesized a series of 6-(substitutedphenyl)-*N'*-((*E*)-(substitutedphenyl)methylidene)-2-methylpyridine-3-carbohydrazides (**5a-n**) and evaluated their antibacterial activity. Structures of these compounds were confirmed by standard studies of FTIR, ^1^H NMR, ^13^C NMR, MS and elemental analysis. Antibacterial activity of synthesized molecules was tested against Gram-positive (*S. aureus* and *E. faecalis*) and Gram-negative (*E. coli* and *P. aeruginosa*) bacterial strains. Synthesized compounds showed good antibacterial activity at a lower concentration than standard. Most of the compounds (**5a**, **5c**, **5i**, **5j** and **5n**) are potent against all the tested bacterial strains with MIC values ranging from 1.56-12.5 μg/mL.

## 1 Introduction

Medicinal Chemistry concerns with the understanding of mechanisms of action of drugs. In current years pyridine derivatives have attracted the attention of researchers on account of their broad range of applications in medicine. In this heterocyclic compounds, various pyridines derivatives of commercial interest find application in market areas mainly due to their important biological activity and their use in agricultural products such as herbicides, insecticides, fungicides and in plant growth regulators. Pyridine derivatives exhibited various types of biological activities viz antimicrobial [1, 2], antibacterial [3], antimycobacterial [4], analgesic [5] antiparkinsonian [6], anticonvulsant [7], antitumoral [8], cytotoxic [9], antimalarial [10], antidiabetic [11], pesticidal [12], inhibitory [13] and receptor antagonists [14].

**Fig. 1:**
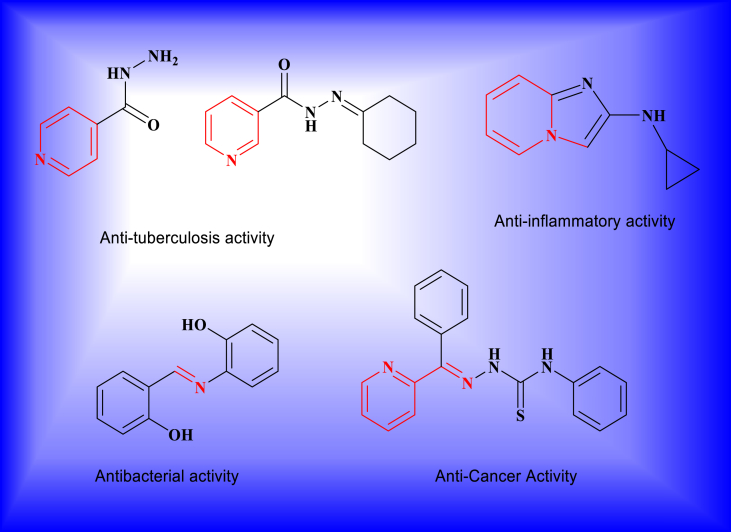
Bioactive pyridinehydrazone and Schiff base derivatives.

Similarly, compounds which contain an azomethine group (R₂C=NR′) is known as Schiff bases. They are formed by the condensation of a primary amine with a carbonyl compound. Their biological properties are due to the presence of imine group. So this is a good intermediate in the reaction involving the interaction of an enzyme with an amino or a carbonyl group of the substrate. In addition to this Schiff base are having excellent biological activities such as antimalarial [15], antibacterial [16], antifungal [17], antiviral [18], antitumor [19] and antioxidant [20] activities. At the same time they have stronger binding affinity towards DNA double helix [21]. Literature reports for pyridinehydrazone and Schiff base analogs as anti-tuberculosis, antimicrobial, anti-inflammatory anticancer agents is presented in Fig. 1 [22].

It was observed that by combining the two biological active pharmacophores we can generate novel molecular templates which will definitely exhibit interesting biological properties. In view of the above findings, an amalgamation of two biologically versatile scaffolds like pyridinehydrazone and azomethine group (-CH = N-) in a single molecular frame was undertaken, hoping that these compounds might possess certain antimicrobial activity. Herein, a variety of aryl pyridine derivatives bearing the azomethine group containing a diverse set of aromatic substitutions were designed and synthesized and evaluated for their antibacterial activity.

## 2 Results and discussion

### 2.1 Chemistry

**Fig. 2:**
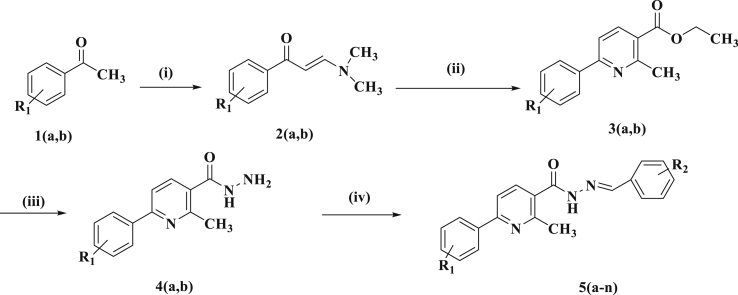
Outline for the synthesis. Reagents and conditions: (i) DMF-DMA, 90 °C, 5–6 h (ii) Ethyl acetoacetate, NH_4_OAc, AcOH, reflux, 3 h (iii) N_2_H_4_, reflux, 5–6 h (iv) Substituted aromatic aldehydes, AcOH, ethanol, reflux, 5 h.

The synthetic path for aimed compounds (5a–n) is depicted in (Fig. 2). The starting material enaminone (2a–b) were synthesized in good yield by commercially available 3,4-dichloroacetophenone and 3,4-dimethoxyacetophenone (1a–b) refluxing with N,N-dimethylformamide dimethyl acetal and these were subsequently converted into ethyl-6-(3,4-dichloro/3,4-dimethoxyphenyl)-2-methylpyridine-3-carboxylate (3a–b) by treating with ethylacetoacetate in presence of ammonium acetate in glacial acetic acid media. Then followed by reaction with hydrazine hydrate in absolute alcohol under reflux condition led to the formation of (4a–b) in good yield. The resultant 6-(3,4-dichloro/3,4-dimethoxyphenyl)-2-methylpyridine-3-carbohydrazide (4a-b) was further converted into corresponding Schiff bases (5a-n) by condensing 6-(3,4-dichloro/3,4-dimethoxyphenyl)-2-methylpyridine-3-carbohydrazide (4a-b) with different substituted benzaldehydes in alcohol by adding the catalytical amount of glacial acetic acid. The reaction mixture was refluxed for 5 h. After completion (TLC), the reaction mixture was cooled and poured into crushed ice, then precipitated solid was filtered, dried and recrystallized from ethanol to get final compounds 5 (a-n) with 88–94% yield. The newly synthesized aimed compounds were confirmed by FTIR, ^1^H NMR, ^13^C NMR, MS and elemental data. The physical properties of synthesized compounds are listed below in Table 1.

**Table 1:** Physical data of the newly synthesized 6-(3,4-dichlorophenyl)-*N'*-((*E*)-(substituted phenyl)methylidene)-2-methylpyridine-3-carbohydrazide (**5a-g**) and *N'*-((*E*)-(substituted phenyl)methylidene)-6-(3,4-dimethoxyphenyl)-2-methylpyridine-3-carbohydrazide (**5h-n**) via above scheme shown in Fig. 2.

Compoud	R^1^	R^2^	Mol.formula	Mol.weight	Yield (%)	M.p (°C)
5a	3,4-Cl_2_	3,4,5-(OMe)_3_	C_23_H_21_Cl_2_N_3_O_4_	473.34	92	150–152
5b	3,4-Cl_2_	2,4-Cl_2_	C_20_H_13_Cl_4_N_3_O	453.15	94	156–158
5c	3,4-Cl_2_	3,4-(OH)_2_	C_20_H_15_Cl_2_N_3_O_3_	416.26	89	182–184
5d	3,4-Cl_2_	3-Br-4-OH-5-OMe	C_21_H_16_BrCl_2_N_3_O_3_	509.18	93	180–182
5e	3,4-Cl_2_	3,4-(OMe)_2_	C_21_H_19_Cl_2_N_3_O_3_	444.31	94	142–144
5f	3,4-Cl_2_	4-OH	C_20_H_15_Cl_2_N_3_O_2_	400.26	90	212–214
5g	3,4-Cl_2_	4-Me	C_21_H_17_Cl_2_N_3_O	398.29	90	110–112
5h	3,4-(OMe)_2_	3,4,5-(OMe)_3_	C_25_H_27_N_3_O_6_	465.5	94	172–174
5i	3,4-(OMe)_2_	2,4-Cl_2_	C_22_H_19_Cl_2_N_3_O_3_	444.31	92	160–162
5j	3,4-(OMe)_2_	3,4-(OH)_2_	C_22_H_21_N_3_O_5_	407.42	92	112–114
5k	3,4-(OMe)_2_	3-Br-4-OH-5-OMe	C_23_H_22_BrN_3_O_5_	500.34	88	124–126
5l	3,4-(OMe)_2_	3,4 -(OMe)_2_	C_24_H_25_N_3_O_5_	435.47	91	176–178
5m	3,4-(OMe)_2_	4-OH	C_22_H_21_N_3_O_4_	391.42	91	202–204
5n	3,4-(OMe)_2_	4-Me	C_23_H_23_N_3_O_3_	389.45	91	186–188

The structure of the synthesized compounds was confirmed by spectral data. According to the FTIR spectroscopic data of the compound **5e**, they exhibited a characteristic peak at 3214 cm^−1^ correspondings to –NH functional group. Absorption bands for C=O and C=N groups were observed at 1651 and 1547 cm^−1^ respectively. In ^1^H NMR spectrum of compound **5e**, showed a sharp singlet at δ 2.68 ppm which indicated the presence of methyl group on pyridine unit. The signal resonating at δ 3.82 ppm was assigned to six protons of the methoxy groups. Also ^1^H NMR spectrum of compound **5e** showed a doublet at δ 6.81 ppm with a coupling constant of 1.5 Hz was assigned to the aromatic proton of the 3,4-dimethoxyphenylgroup. The signals resonating in the range of δ 6.85–6.89 ppm and δ 7.99–8.03 ppm as multiplets due to three protons supported the presence of aryl nucleus in the molecule. The signals resonating at δ 7.43 and 7.67 ppm as two doublets with coupling constant 7.5 Hz were assigned to aromatic protons. Remaining aromatic protons resonated at 7.64 ppm as a singlet and in the range of δ 7.80–7.82 ppm as a doublet of doublet with coupling constants 1.5 Hz and 7.5 Hz. Furthermore, –NH proton appeared at δ 8.95 ppm as a broad signal which in turn confirmed the formation of the target molecule [23]. The structure of new Schiff base **5e** was also confirmed by ^13^C NMR spectrum. In ^13^C NMR spectrum, methyl and methoxy carbons appeared as different singlets at 25.6, 55.9 and 60.0 ppm respectively. The peak observed at 161.1 ppm was corresponding to C=*N group* of the compound. In ^13^C NMR spectrum, C=O and C–N carbons peaks were observed at 167.8 and 169.5 ppm. The aromatic carbons appeared in the range of 108.8–154.1 ppm. The mass spectrum provided support to the structure of the compound **5e** as it displayed (M+1) (M+3) and (M+4) ion peaks at m/z 444.16, 446.10 and 447.08, respectively, which is in agreement with its molecular formula C_21_H_19_Cl_2_N_3_O_3_, evidencing the formation of compound. The elemental analysis also gave satisfactory values for the percentage of C, H, and N present in the synthesized molecule, and is presented in the experimental section.

The formation of new Schiff base compound **5k** was also confirmed FTIR, mass, ^1^H, ^13^C NMR and elemental analyses. The FTIR information precise functional group confirmation for the arrangement of the predictable structures. A band found at the frequency of 3366 cm^−1^ confirmed that the –NH group present in the compound. And FTIR spectral data showed the presence of carbonyl (-C=O) and C=N groups bands at the of the frequency of 1645 and 1581 cm^−1^ respectively. The stretching vibrations at 699 cm^−1^ correspond to -C-Br bonds, which supported the product formation. ^1^H and ^13^C NMR spectral data of the title compound **5k** gave satisfactory results. ^1^H NMR spectrum of the representative compound showed a singlet for the methyl protons at δ 2.67 ppm. Nine methoxy protons appeared as two distant singlet at δ 3.81 and δ 3.83 ppm. A characteristic singlet at δ 4.58 ppm was assigned to hydroxyl proton of the derivative. Aromatic protons appeared in the range of δ 6.78–8.01 ppm. A broad singlet at δ 8.99 ppm evidences the presence of the N–*H group* [23]. Similarly, ^13^C NMR also justified product formation by giving the expected peaks. The signals at δ 177.3 and 174.7 ppm were observed for the = C–N- and –C=O carbons respectively. In the ^13^C NMR the presence of peaks at δ 168.8 (C–O) and δ 163.8 (-C=N) also confirmed the formation of the product. The remaining aromatic carbons were found in the range of δ 108.0–162.7 ppm. Methoxy carbons appeared as different singlets at δ 56.0, 56.3 and 56.9 ppm. A signal at δ 23.6 ppm was observed for the methyl carbon of the compound. The mass spectrum (ESI-MS) of **5k** shows a protonated molecular ion peak (M-H)^-^ with m/z value of 498.0 and (M+2H)^+^ with m/z value of 501.01 which confirms the formation of the product and the elemental analysis data were in full agreement with its molecular formula C_23_H_22_BrN_3_O_5_. The physical data of the synthesized derivatives (**5a-n**) is presented in Table 1.

### 2.2 Antibacterial assay

**Fig. 3:**
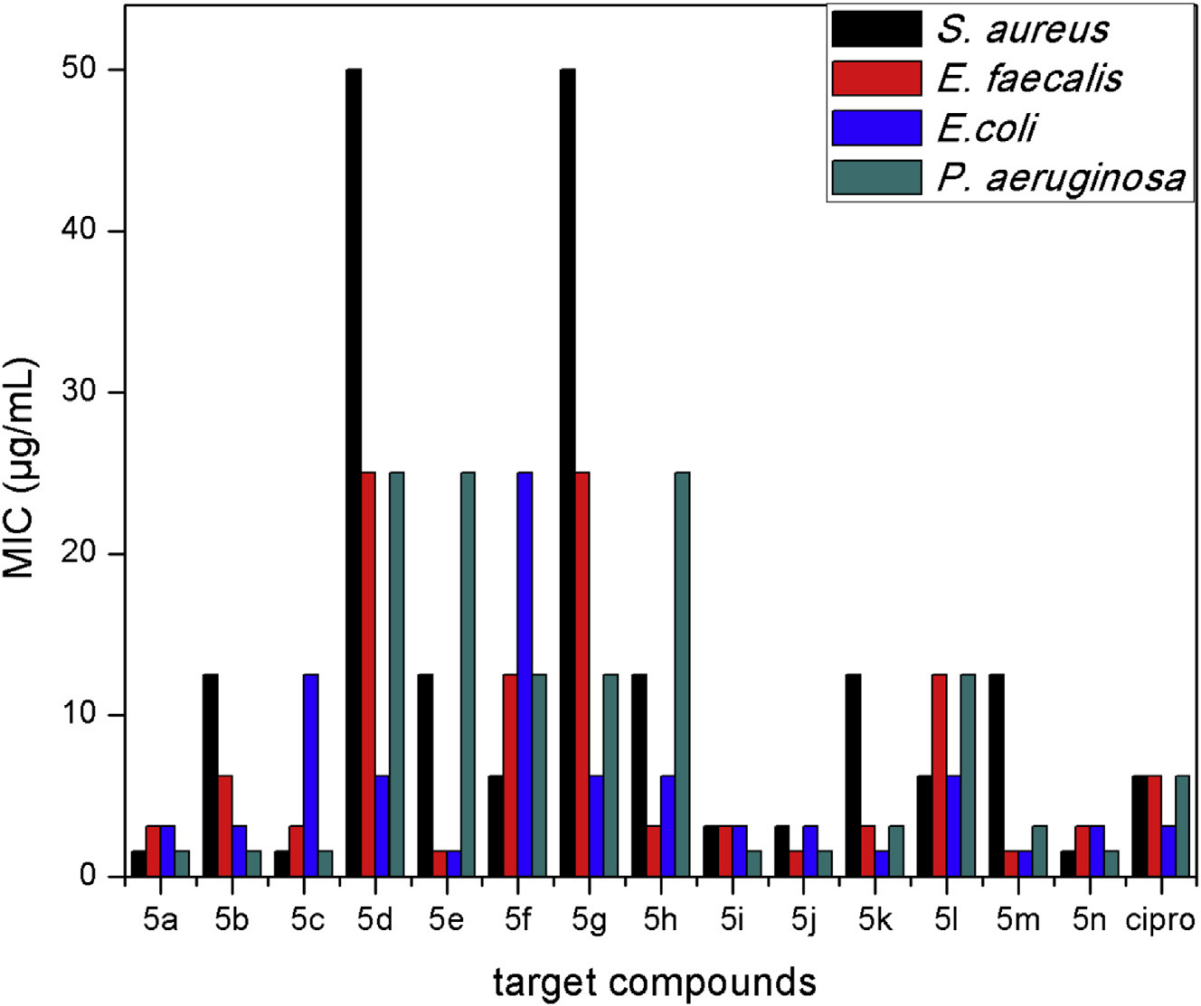
Graphical representation of antibacterial studies of the target compounds against four bacterial strains.

All the synthesized compounds were evaluated for their antibacterial activity using the broth dilution method [24]. Antibacterial activity was evaluated against two Gram-positive (*S. aureus* and *E. faecalis*) and two Gram-negative (*E. coli* and *P. aeruginosa*) bacterial strains. Ciprofloxacin was used as a standard. The results of the preliminary antibacterial activity of compounds **5(a-n)** are presented in Table 2. Graphical representation of results of antibacterial studies of the target compounds against four bacterial strains is presented in Fig. 3.

**Table 2:** Antibacterial activity of synthesized compounds (**5a-n**) by broth dilution method.

Antibacterial activity data of the target compounds (5a-n) in terms of Minimum inhibitory concentration in μg/mL (MIC)						
Entry	R^1^	R^2^	Gram positive		Gram negative	
			*S. aureus*	*E. faecalis*	*E. coli*	*P. aeruginosa*
5a	3,4-Cl_2_	3,4,5-(OMe)_3_	**1.56**	**3.125**	**3.125**	**1.56**
5b	3,4-Cl_2_	2,4-Cl_2_	12.5	6.25	3.125	**1.56**
5c	3,4-Cl_2_	3,4-(OH)_2_	**1.56**	**3.125**	**12.5**	**1.56**
5d	3,4-Cl_2_	3-Br-4-OH-5-OMe	50	25	6.25	25
5e	3,4-Cl_2_	3,4-(OMe)_2_	12.5	**1.56**	**1.56**	25
5f	3,4-Cl_2_	4-OH	6.25	12.5	25	12.5
5g	3,4-Cl_2_	4-Me	50	25	6.25	12.5
5h	3,4-(OMe)_2_	3,4,5-(OMe)_3_	12.5	3.125	6.25	25
5i	3,4-(OMe)_2_	2,4-Cl_2_	**3.125**	**3.125**	**3.125**	**1.56**
5j	3,4-(OMe)_2_	3,4-(OH)_2_	**3.125**	**1.56**	**3.125**	**1.56**
5k	3,4-(OMe)_2_	3-Br-4-OH-5-OMe	12.5	**3.125**	**1.56**	**3.125**
5l	3,4-(OMe)_2_	3,4 -(OMe)_2_	6.25	12.5	6.25	12.5
5m	3,4-(OMe)_2_	4-OH	12.5	**1.56**	**1.56**	**3.125**
5n	3,4-(OMe)_2_	4-Me	**1.56**	**3.125**	**3.125**	**1.56**
Ciprofloxacin			6.25	6.25	3.125	6.25

Bold represents that in minimum concentration the synthesized compounds are active.

The minimum inhibitory concentration (MIC) of the synthesized derivatives were compared with standard ciprofloxacin, it revealed that almost all the newly synthesized compounds showed excellent antibacterial activity against both Gram-positive (*S. aureus* and *E. faecalis)* and Gram-negative (*E. coli* and *P. aeruginosa*) bacterial strains. From the screening results, it could be seen that the majority of the tested compounds displayed excellent antibacterial activity [minimum inhibitory concentration (MIC) values in the range of 1.56–12.5 μg/mL]. Result of antibacterial screening proved that Schiff Bases containing arylpyrimidines can be good antibacterial agents.

Compounds **5a**, **5c**, **5i**, **5j** and **5n** belonging to the methylpyridine-3-carbohydrazides series exhibited best antibacterial potency against four bacterial strains with minimum inhibitory concentration (MIC) values of 1.56–3.125 μg/mL. Compounds **5a** (R^1^-3,4-dichlorophenyl, R^2^-3,4,5-trimethoxyphenyl), **5c** (R^1^-3,4-dichlorophenyl, R^2^-3,4-dihydroxyphenyl), **5i** (R^1^-3,4-dimethoxyphenyl, R^2^-2,4-dichlorophenyl), **5j** (R^1^-3,4-dimethoxyphenyl, R^2^-3,4-dihydroxyphenyl) and **5n** (R^1^-3,4-dimethoxyphenyl, R^2^-4-methylphenyl) have shown significant activity at lower concentration than standard drug. Compound **5a** which is a combination of dichloro and trimethoxy substituted derivative showed MIC value of 1.56, 3.125, 3.125 and 1.56 μg/mL against *S. aureus*, *E. faecalis*, *E. coli* and *P. aeruginosa* respectively. When trimethoxy is replaced by dihydroxy substituted benzaldehyde the inhibition activity of the compound **5c** remained almost same except in case of *E. coli*. It showed MIC value of 1.56, 3.125, 12.5 and 1.56 μg/mL against *S. aureus*, *E. faecalis*, *E. coli* and *P. aeruginosa* respectively. Compounds **5i** (R^1^-3,4-dimethoxyphenyl, R^2^-2,4-dichlorophenyl), **5j** (R^1^-3,4-dimethoxyphenyl, R^2^-3,4-dihydroxyphenyl) and **5n** (R^1^-3,4-dimethoxyphenyl, R^2^-4-methylphenyl) which are combination of electron donating groups showed high activity with minimum inhibitory concentration (MIC) in the range of 1.56–3.125 μg/mL against both Gram-positive (*S. aureus* and *E. faecalis)* and Gram-negative (*E. coli* and *P. aeruginosa*) bacterial strains. All MIC values with the respective substitution on the aryl group is represented in Table 2.

Compound **5b** (R^1^-3,4-dichlorophenyl, R^2^-2,4-dichlorophenyl) showed MIC value of 1.56 μg/mL against *P. aeruginosa* (Gram negative) but failed to show activity against other bacterial strains. Compound **5e** (R^1^-3,4-dichlorophenyl, R^2^-3,4-dimethoxyphenyl) produced good inhibiting potency toward *E. faecalis* (Gram-positive) *E. coli* (Gram negative) with the MIC value of 1.56 μg/mL, while the compounds **5f**, **5g**, **5h** were moderately active against the tested organisms. Furthermore, compound **5k** (R^1^-3,4-dimethoxyphenyl, R^2^-3-bromo-4-hydroxy-5-methoxyphenyl) produced marked activity at low concentration against *E. faecalis*, *E. coli* and *P. aeruginosa* with MIC value of 3.125, 1.56 and 3.125 μg/mL respectively. For compound **5m** (R^1^-3,4-dimethoxyphenyl, R^2^-4-hydroxyphenyl) was demonstrated to be a potent molecule against *E. faecalis* (Gram-positive) and *E. coli* (Gram-negative) with MIC values of 1.56 μg/mL.

## 3 Conclusions

In conclusion, a series of 6-(substitutedphenyl)-*N'*-((*E*)-(substitutedphenyl)methylidene)-2-methylpyridine-3-carbohydrazides (**5a–n**) were prepared in good yield and their antibacterial activity was investigated. Evaluations of the antibacterial activity of the derivatives were carried out by following the broth dilution method. Results acquired from the antibacterial screening showed that most of the compounds (**5a**, **5c**, **5i**, **5j** and **5n**) are potent against bacterial strains. Halogen, methoxy, hydroxy and methyl substituted derivatives good inhibition against the tested bacterial strains when compared to standard. From the antimicrobial activity results, the structure.

activity relationship can be deduced for the test compounds (**5a-n**). From the results, it is clear that electron donating groups increased the inhibition and showed significant results in minimum concentration. Based on the results obtained in the present study, it is concluded that design was successful in generating members of the antibacterial agent family.

## 4 Experimental

### 4.1 Materials and methods

All the essential chemicals used for this synthesis were procured from Merck, S.D. Fine, Sigma-Aldrich Company. The reagents and solvents were analytical grade and used without further purification. Melting points of newly synthesized compounds was determined via the open capillary technique. and was uncorrected. The complection of every steps was identified by TLC analyses on silica plates and spots were visualised via UV light, exposure to iodine vapours. Hexane:Ethyl acetate (3:1) were the adopted solvent system. FTIR spectra were recorded on Shimadzu FTIR infrared spectrometer in KBr pellets also wavenumbers are specified in cm^−1^. ^1^H NMR and ^13^C NMR spectra were recorded on Bruker Avance III, 400 MHz and 100 MHz spectrometers, in the CDCl_3_ solvent system and chemical shift values were expressed in parts per million (ppm) downfield from TMS, an internal standard. Mass spectra recorded on Water, synapt G2 high detection mass spectrometry and are uncorrected. Elemental analysis was carried out using CHNS Elementar Vario EL III. Compounds were prepared according to reported procedures.

### 4.2 General synthetic procedure for 1-(3,4-dichloro/3,4-dimethoxyphenyl)-3-(dimethylamino)prop-2-en-1-one 2(a,b)

In the first synthetic step commercially available substituted acetophenones (1a–b 1 mmol) was refluxed with N,N-dimethylformamide dimethyl acetal (1 mmol) at 90 °C for 5–6 h. After complection of the reaction, the reaction mixture was extracted with dichloromethane. The excess solvent was removed under reduced pressure. The liquid product 1-(substitutedphenyl)-3-(dimethylamino)prop-2-en-1-one 2 (a,b) was obtained in 90% yield [25].

### 4.3 General synthetic procedure for ethyl 6-(3,4-dichloro/3,4-dimethoxyphenyl)-2-methylpyridine-3-carboxylate 3(a,b)

Ethylaectoacetate (1.1 mmol) and ammonium acetate (4 mmol) were added to the solution of appropriate enaminone (2a–b) (1 mmol) in glacial acetic acid (5 mmol). The reaction mixture was heated under reflux for 3 h and monitored by using TLC. After completion of the reaction, the reaction mixture was allowed to cool to room temperature, and the solution was poured into ice-cold water, the residue obtained was filtered, and washed with hexane followed by water, recrystallized from ethanol [25].

### 4.4 General synthetic procedure for 6-(3,4-dichloro/3,4-dimethoxyphenyl)-2-methylpyridine-3-carbohydrazide 4(a,b)

A mixture of ethyl 6-(substitutedphenyl)-2-methylpyridine-3-carboxylate (3a,b 5 mmol) and hydrazine hydrate (5 mmol) in ethanol was refluxed for 4 h, cooled to room temperature and filtered. The excess solvent was then distilled off under reduced pressure, and the concentrated solution was quenched in ice-cold water. The separated solid was filtered, washed and dried. The crude product was purified by recrystallization from ethanol to give the desired product (4a,b) as white solid [25].

### 4.5 General synthetic procedure for 6-(3,4-dichloro/3,4-dimethoxyphenyl)-*N*'-[(*E*)-(substitutedphenyl)methylidene]-2-methylpyridine-3-carbohydrazide 5(a–n)

A mixture of compound (4a,b 10 mmol) and substituted aldehydes (10 mmol) in 10 mL of absolute ethanol in presence of catalytic amount of acetic acid was refluxed for 5 h. After cooling, the precipitated product was filtered, washed with water and dried. The solid thus formed on cooling was recrystallized from ethanol to give Schiff base 5 (a–n).

#### 4.5.1 6-(3,4-dichlorophenyl)-2-methyl-N'-((E)-(3,4,5-trimethoxyphenyl)methylidene)pyridine-3-carbohydrazide (5a)

Yellow solid (yield 92%), m. p. 150–152 °C; FTIR (ATR) (cm^−1^): 3214 (NH-sharp), 1651 (C=O), 1547 (C=N); ^1^H NMR (400 MHz, CDCl_3_, δ ppm): 2.56 (3H, s, –CH_3_), 3.71 (6H, s, –OCH_3_), 3.83 (3H, s, –OCH_3_), 7.22 (2H, d, *J* = 7.5 Hz, Ar–H), 7.55 (2H, d, *J* = 7.5 Hz, Ar–H), 7.89–7.91 (2H, m, Ar–H), 8.25–8.30 (2H, m, Ar–H), 8.47 (1H, s, –CH), 8.95 (1H, s, –NH); ^13^C NMR (100 MHz, CDCl_3_, δ ppm): 22.2, 56.1, 60.8, 104.0, 122.5, 127.1, 128.0, 128.2, 128.9, 130.7, 132.0, 132.7, 135.7, 138.4, 141.5, 146.8, 152.7, 153.2, 163.2, 165.8; EI-MS: m/z (M+1, 474.09; M+ 2, 475.09); Anal. calcd for C_23_H_21_Cl_2_N_3_O_4_: C, 58.24; H, 4. 46; N, 08.86. Found: C, 58.27; H, 4. 44; N, 08.84.

#### 4.5.2 6-(3,4-dichlorophenyl)-N'-((E)-(2,4-dichlorophenyl)methylidene)-2-methylpyridine-3-carbohydrazide (5b)

Yellow solid (yield 94%), m. p. 156–158 °C; FTIR (ATR) (cm^−1^): 3214 (NH-sharp), 1651 (C=O), 1547 (C=N); ^1^H NMR (400 MHz, CDCl_3_, δ ppm): 2.65 (3H, s, –CH_3_), 7.51–7.55 (2H, m, Ar–H),7.70 (1H, s, Ar–H), 7.89–8.00 (3H, m, Ar–H), 8.25–8.30 (3H, m, Ar–H), 8.47 (1H, s, –CH), 8.95 (1H, s, –NH); ^13^C NMR (100 MHz, CDCl_3_, δ ppm): 22.2, 122.5, 127.0, 127.1, 128.2, 128.3, 128.9, 129.0, 129.1, 130.7, 131.0, 132, 132.7, 132.8, 135.7, 138.4, 138.7, 152.7, 163.2, 165.8; EI-MS: m/z (M+1, 454.98); Anal. calcd for C_20_H_13_Cl_4_N_3_O: C, 53.01; H, 2.89; N, 9.27; Found: C, 53.03; H, 2.90; N, 9.35.

#### 4.5.3 6-(3,4-dichlorophenyl)-N'-((E)-(3,4-dihydroxyphenyl)methylidene)-2-methylpyridine-3-carbohydrazide (5c)

Brown solid (yield 89%), m. p. 182–184 °C; FTIR (ATR) (cm^−1^): 3214 (NH-sharp), 1651 (C=O), 1547 (C=N); ^1^H NMR (400 MHz, CDCl_3_, d ppm): ^1^H NMR (400 MHz, CDCl_3_, δ ppm): 2.56 (3H, s, –CH_3_), 5.00 (2H, s), 6.71 (1H, d, *J* = 7.5, Hz, Ar–H), 7.21–7.23 (2H, m, Ar–H), 7.55 (1H, d, *J* = 7.5 Hz), 7.89–7.91 (2H, m, Ar–H), 8.25–8.30 (3H, m, Ar–H), 8.47 (1H, s, –CH), 8.95 (1H, s, –NH); ^13^C NMR (100 MHz, CDCl_3_, δ ppm): 22.2, 116.3, 117.4, 122.5, 123.2, 127.1, 128.2, 128.9, 130.7, 131.3, 132, 132.7, 135.7, 138.4, 146.1, 146.8, 149.6, 152.7, 163.2, 165.8; EI-MS: m/z (M+1, 416.05; M+2, 417.05); Anal. calcd for C_20_H_15_Cl_2_N_3_O_3_: C, 57.71; H, 3.63; N, 10.09. Found: C, 57.75; H, 3.66; N10.15.

#### 4.5.4 N'-((E)-(3-bromo-4-hydroxy-5-methoxyphenyl)methylidene)-6-(3,4-dichlorophenyl)-2-methylpyridine-3-carbohydrazide (5d)

Yellow solid (yield 93%), m. p. 180–182 °C; FTIR (ATR) (cm^−1^): 3214 (NH-sharp), 1651 (C=O), 1547 (C=N); ^1^H NMR (400 MHz, CDCl_3_, d ppm): ^1^H NMR (400 MHz, CDCl_3_, δ ppm): 2.56 (3H, s, –CH_3_), 3.83 (3H, s, –OCH_3_), 5.00 (1H, s, –OH), 7.31 (1H, s, Ar–H), 7.42 (1H, s, Ar–H), 7.55 (1H, d, *J* = 7.5 Hz, Ar–H), 7.89–7.91 (2H, m, Ar–H), 8.25–8.30 (3H, m, Ar–H), 8.47 (1H, s, –CH), 8.95 (1H, s, –NH); ^13^C NMR (100 MHz, CDCl_3_, δ ppm): ^13^C NMR (100 MHz, CDCl_3_, d ppm): 22.2, 56.1, 111.1, 114.9, 122.5, 122.6, 127.1, 128.2, 128.9, 129.5, 130.7, 132, 132.7, 135.7, 138.4, 144.1, 146.8, 152.7, 153.7, 163.2, 165.8; EI-MS: m/z (M+1, 508.97; M+2, 510.95); Anal. calcd for C_21_H_16_BrCl_2_N_3_O_3_: C, 49.54; H, 3.17; N, 8.25. Found: C, 49.57; H, 3.19; N10.19.

#### 4.5.5 6-(3,4-dichlorophenyl)-N'-((E)-(3,4-dimethoxyphenyl)methylidene)-2-methylpyridine-3-carbohydrazide (5e)

Yellow solid (yield 94%), m. p. 142–144 °C; FTIR (ATR) (cm^−1^): 3214 (NH-sharp), 1651 (C=O), 1547 (C=N); ^1^H NMR (400 MHz, CDCl_3_, δ ppm): 2.68 (3H, s, –CH_3_), 3.82 (6H, s, –OCH_3_), 6.81 (1H, d, *J* = 1.5 Hz, Ar–H), 6.85–6.89 (2H, m, Ar–H), 7.43 (1H, d, *J* = 7.5 Hz, Ar–H), 7.64 (1H, s, Ar–H), 7.67 (1H, d, *J* = 7.5 Hz), 7.80–7.82 (1H, dd, *J* = 7.5, 1.5 Hz, Ar–H), 7.99–8.03 (1H, m, Ar–H), 8.04 (1H, s, Ar–H), 8.95 (1H, s, –NH); ^13^C NMR (100 MHz, CDCl_3_, δ ppm): 25.6, 55.9, 60.0, 108.8, 110.7, 116.5, 123.9, 126.1, 127.3, 128.9, 130.7, 145.3, 149.4, 151.8, 154.1, 161.1, 167.8, 169.5; EI-MS: m/z (M+1, 444.16; M+2, 446.10); Anal. calcd for C_21_H_19_Cl_2_N_3_O_3_: C, 59.47; H, 4.31; N, 9.46. Found: C, 59.49; H, 4.35; N, 9.49.

#### 4.5.6 6-(3,4-dichlorophenyl)-N'-((E)-(4-hydroxyphenyl)methylidene)-2-methylpyridine-3-carbohydrazide (5f)

Yellow solid (yield 90%), m. p. 212–214 °C; FTIR (ATR) (cm^−1^): 3334 (NH-sharp), 1594 (C=O), 1509 (C=N); ^1^H NMR (400 MHz, CDCl_3_, δ ppm): 2.56 (3H, s, –CH_3_), 5.00 (1H, s, –OH), 6.85 (2H, d, *J* = 8 Hz Ar–H), 7.55–7.66 (3H, m, Ar–H), 7.89–7.91 (3H, m, Ar–H), 8.25–8.30 (3H, m, Ar–H), 8.47 (1H, s, –CH), 8.95 (1H, s, –NH); ^13^C NMR (100 MHz, CDCl_3_, δ ppm): 22.2, 116.0, 122.5, 126.3, 127.1, 128.2, 128.9, 130.6, 130.7, 132.0, 132.7, 135.7, 138.4, 146.8, 152.7, 160.8, 163.2, 165.8; EI-MS: m/z (M+1, 400.06; M+2, 401.05); Anal. calcd for C_20_H_15_Cl_2_N_3_O_2_: C, 60.01; H, 3.78; N, 10.50. Found: C, 60.05; H, 3.81; N, 10.52.

#### 4.5.7 6-(3,4-dichlorophenyl)-2-methyl-N'-((E)-(4-methylphenyl)methylidene)pyridine-3-carbohydrazide (5g)

Yellow solid (yield 90%), m. p. 110–112 °C; FTIR (ATR) (cm^−1^): 3214 (NH-sharp), 1651 (C=O), 1547 (C=N); ^1^H NMR (400 MHz, CDCl_3_, δ ppm): 2.41 (3H, s, –CH_3_), 2.56 (3H, s, –CH_3_), 7.40 (2H, d, *J* = 7.5 Hz, Ar–H), 7.55 (1H, s, Ar–H), 7.89–7.91 (4H, m, Ar–H), 8.25–8.30 (3H, m, Ar–H), 8.47 (1H, s, –CH), 8.95 (1H, s, –NH); ^13^C NMR (100 MHz, CDCl_3_, δ ppm): 21.3, 22.2, 122.5, 126.1, 127.1, 128.2, 128.9, 129.1, 130.7, 132, 132.7, 135.7, 138.4, 140.7, 146.8, 152.7, 163.2, 165.8; EI-MS: m/z (M+1, 398.08; M+2, 399.07); Anal. calcd for C_21_H_17_Cl_2_N_3_O: C, 63.33; H, 4.30; N, 10.55. Found: C, 63.35; H, 4.32; N, 10.56.

#### 4.5.8 6-(3,4-dimethoxyphenyl)-2-methyl-N'-((E)-(3,4,5-trimethoxyphenyl)methylidene)pyridine-3-carbohydrazide (5h)

Yellow solid (yield 94%), m. p. 172–174 °C; FTIR (ATR) (cm^−1^): 3184 (NH-sharp), 1649 (C=O), 1580(C=N); ^1^H NMR (400 MHz, CDCl_3_, δ ppm): 2.56 (3H, s, –CH_3_), 3.71 (3H, s, –OCH_3_), 3.83 (9H, s, –OCH_3_), 3.92 (3H, s, –OCH_3_), 7.00 (1H, d, *J* = 7.5 Hz, Ar–H), 7.22 (2H, d, *J* = 7.5 Hz, Ar–H), 7.62–7.68 (2H, m, Ar–H), 7.89–7.91 (2H, m, Ar–H), 8.47 (1H, s, –CH), 8.95 (1H, s, –NH); ^13^C NMR (100 MHz, CDCl_3_, δ ppm): 22.2, 56.1, 60.8, 104.0, 108.5, 111, 120.9, 122.5, 128, 128.2, 133.4, 138.4, 141.5, 146.8, 148.4, 150.3, 152.7, 153.2, 163.2, 165.8; EI-MS: m/z (M+1, 466.19; M+2, 467.20); Anal. calcd for C_25_H_27_N_3_O_6_: C, 64.50; H, 5.85; N, 9.03. Found: C, 64.54; H, 5.83; N, 9.07.

#### 4.5.9 N'-((E)-(2,4-dichlorophenyl)methylidene)-6-(3,4-dimethoxyphenyl)-2-methylpyridine-3-carbohydrazide (5i)

Yellow solid (yield 92%), m. p. 160–162 °C; IR (ATR) (cm^−1^): 3184 (NH-sharp), 1649 (C=O), 1580 (C=N); ^1^H NMR (400 MHz, CDCl_3_, δ ppm): 2.56 (3H, s, –CH_3_), 3.91 (3H, s, –OCH_3_), 4.01 (3H, s, –OCH_3_), 6.97 (2H, d, *J* = 7.5 Hz), 7.32–7.59 (3H, m, Ar–H), 7.78 (2H, d, *J* = 7.5 Hz, Ar–H), 8.15–8.20 (2H, m, Ar–H), 9.23 (1H, s, –NH); ^13^C NMR (100 MHz, CDCl_3_, δ ppm): 23.6, 56.0, 56.1, 105.7, 110.2, 111.2, 116.3, 119.9, 127.8, 129.7, 133.7, 138.3, 141.3, 149.4, 153.8, 156.7, 160.2, 170.7, 172.4; EI-MS: m/z (M+1, 444.08; M+2, 445.08); Anal. calcd for C_22_H_19_Cl_2_N_3_O_3_: C, 59.47; H, 4.31; N, 9.46. Found: C, 59.49; H, 4.37; N, 9.49.

#### 4.5.10 N'-((E)-(3,4-dihydroxyphenyl)menthylidene)-6-(3,4-dimethoxyphenyl)-2-methylpyridine-3-carbohydrazide (5j)

Brown solid (yield 92%), m. p. 112–114 °C; FTIR (ATR) (cm^−1^): 3184 (NH-sharp), 1649 (C=O), 1580(C=N); ^1^H NMR (400 MHz, CDCl_3_, δ ppm): 2.56 (3H, s, –CH_3_), 3.83 (3H, s, –OCH_3_), 3.92 (3H, s, –OCH_3_), 5.00 (2H, s –OH), 6.71 (1H, d, *J* = 7.5 Hz, Ar–H), 7.00 (1H, d, *J* = 7.5 Hz, Ar–H), 7.22–7.23 (2H, m, Ar–H), 7.62–7.68 (2H, m, Ar–H), 7.89–7.91 (2H, m, Ar–H), 8.47 (1H, s, –CH), 8.95 (1H, s, –NH); ^13^C NMR (100 MHz, CDCl_3_, δ ppm): 22.2, 56.1, 108.5, 111, 116.3, 117.4, 120.9, 122.5, 123.2, 128.2, 131.3, 133.4, 138.4, 146.1, 146.8, 148.4, 150.3, 152.7, 163.2, 165.8; EI-MS: m/z (M+1,408.15; M+2, 409.15); Anal. calcd for C_22_H_21_N_3_O_5_: C, 64.86; H, 5.20; N, 10.31. Found: C, 64.89; H, 5.25; N, 10.33.

#### 4.5.11 N'-((E)-(3-bromo-4-hydroxy-5-methoxyphenyl)methylidene)-6-(3,4-dimethoxyphenyl)-2-methylpyridine-3-carbohydrazide (5k)

Yellow solid (yield 88%), m. p. 124–126 °C; FTIR (ATR) (cm^−1^): 3366 (NH-sharp), 1645 (C=O), 1581 (C=N), and 699 (C–Br); ^1^H NMR (400 MHz, CDCl_3_, δ ppm): 2.67 (3H, s, –CH_3_), 3.81 (3H, s, –OCH_3_), 3.83 (6H, s, –OCH_3_), 4.58 (1H, s –OH), 6.78 (1H, d, *J* = 1.5 Hz, Ar–H), 7.00 (1H, d, *J* = 7.5, Hz, Ar–H), 7.16 (1H, d, *J* = 1.5 Hz, Ar–H), 7.42–7.45 (2H, m, Ar–H), 7.61 (1H, d, *J* = 7.5 Hz, Ar–H), 7.68 (1H, s, Ar–H), 8.01 (1H, d, *J* = 7.5 Hz, Ar–H), 8.99 (1H, s, –NH); ^13^C NMR (100 MHz, CDCl_3_, δ ppm): 23.6, 56.0, 56.3, 56.9, 108.0, 108.2, 123.5, 125.7, 128.6, 130.1, 139.5, 142.9, 147.7, 148.8, 153.6, 157.9, 159.6, 162.7, 163.8, 168.8, 174.7, 177.3; EI-MS: m/z (M-1, 498.00; M+2, 501.01); Anal. calcd for C_23_H_22_BrN_3_O_5_: C, 55.21; H, 4.43; N, 8.40. Found: C, 55.26; H, 4.47; N, 8.42.

#### 4.5.12 6-(3,4-dimethoxyphenyl)-N'-((E)-(3,4-dimethoxyphenyl)methylidene)-2-methylpyridine-3-carbohydrazide (5l)

Yellow solid (yield 91%), m. p. 176–178 °C; FTIR (ATR) (cm^−1^): 3184 (NH-sharp), 1649 (C=O), 1580(C=N); ^1^H NMR (400 MHz, CDCl_3_, δ ppm): 2.56 (3H, s, –CH_3_), 3.83 (6H, s, –OCH_3_), 3.85 (3H, s, –OCH_3_), 3.92 (3H, s, –OCH_3_), 6.98–7.00 (2H, m, Ar–H), 7.23 (1H, d, *J* = 7.5 Hz, Ar–H), 7.56–7.68 (3H, m, Ar–H), 7.89–7.91 (2H, m, Ar–H), 8.47 (1H, s, –CH), 8.95 (1H, s, –NH); ^13^C NMR (100 MHz, CDCl_3_, δ ppm): 22.5, 56.1, 108.5, 109.2, 111, 111.7, 120.9, 122.5, 128.2, 130.6, 133.4, 138.4, 146.8, 148.4, 150.3, 152.1, 152.7, 163.2, 165.8; EI-MS: m/z (M+1,436.18; M+2, 437.19); Anal. calcd for C_24_H_25_N_3_O_5_: C, 66.19; H, 5.79; N, 9.65. Found: C, 66.21; H, 5.82; N, 9.68.

#### 4.5.13 6-(3,4-dimethoxyphenyl)-N'-((E)-(4-hydroxyphenyl)methylidene)-2-methylpyridine-3-carbohydrazide (5m)

Yellow solid (yield 91%), m. p. 202–204 °C; FTIR (ATR) (cm^−1^): 3184 (NH-sharp), 1649 (C=O), 1580(C=N); ^1^H NMR (400 MHz, CDCl_3_, δ ppm): 2.56 (3H, s, –CH_3_), 3.83 (3H, s, –OCH_3_), 3.92 (3H, s, –OCH_3_), 5.00 (1H, s, –OH), 6.85 (2H, d, *J* = 7.5 Hz, Ar–H), 7.00 (1H, d, *J* = 7.5 Hz, Ar–H), 7.62–7.68 (4H, m, Ar–H), 7.89–7.91 (2H, m, Ar–H), 8.47 (1H, s, –CH), 8.95 (1H, s, –NH); ^13^C NMR (100 MHz, CDCl_3_, δ ppm): 22.5, 56.1, 108.5, 111, 116, 120.9, 122.5, 126.3, 128.2, 130.6, 133.4, 138.4, 146.8, 148.4, 150.3, 152.7, 160.8, 163.2, 165.8; EI-MS: m/z (M+1,392.16; M+2, 393.16); Anal. calcd for C_22_H_21_N_3_O_4_: C, 67.51; H, 5.41; N, 10.74. Found: C, 67.55; H, 5.46; N, 10.79.

#### 4.5.14 6-(3,4-dimethoxyphenyl)-2-methyl-N'-((E)-(4-methylphenyl)methylidene)pyridine-3-carbohydrazide (5n)

Yellow solid (yield 91%), m. p. 186–188 °C; FTIR (ATR) (cm^−1^): 3184 (NH-sharp), 1649 (C=O), 1580(C=N); ^1^H NMR (400 MHz, CDCl_3_, δ ppm): 2.41 (3H, s, –CH_3_), 2.56 (3H, s, –CH_3_), 3.83 (3H, s, –OCH_3_), 3.92 (3H, s, –OCH_3_), 7.00 (1H, d, *J* = 7.5 Hz, Ar–H), 7.40 (2H, d, *J* = 7.5 Hz, Ar–H), 7.62–7.68 (2H, m, Ar–H), 7.89–7.91 (2H, m, Ar–H), 8.47 (1H, s, –CH), 8.95 (1H, s, –NH); ^13^C NMR (100 MHz, CDCl_3_, δ ppm): 21.3, 22.2, 56.1, 108.5, 110, 120.9, 122.5, 126.1, 128.2, 129.1, 130.7, 133.4, 138.4, 140.7, 146.8, 148.4, 150.3, 152.7, 163.2, 165.8; EI-MS: m/z (M+1,390.18; M+2, 391.18); Anal. calcd for C_23_H_23_N_3_O_3_: C, 70.93; H, 5.95; N, 10.79. Found: C, 70.98; H, 5.98; N, 10.82.

### 4.6 Antibacterial activity of the compounds

Synthesized novel compounds (**5a-n**) were assayed for their antibacterial activity against four bacterial strains. *Staphylococcus aureus* and *E. faecalis* represented Gram-positive bacterial strains and *E. coli*, and *Pseudomonas aeruginosa* represented Gram-negative bacterial strains. Broth dilution method [24] was followed for the determination of antibacterial activity. Ciprofloxacin was used as a positive control for antibacterial activity. The MIC was defined as the lowest concentration without visible growth. The nine dilutions of each drug (**5a-n**) and Ciprofloxacin have to be done with brain heart infusion (BHI) for MIC. In the initial tube, 2 μL of the drug was added into the 380 μL of BHI broth. For further dilutions, 200 μL of BHI broth was added into the next 9 tubes separately. Then from the initial tube, 200 μL was transferred to the first tube containing 200 μL of BHI broth. This was considered as 10-1 dilution. From 10-1 diluted tube, 200 μL was transferred to the second tube to make 10-2 dilution. The serial dilution was repeated up to 10-9 dilution for each drug. From the maintained stock cultures of essential organisms, 5 μL was taken and added into 2 ml of BHI broth. Successively in each diluted tube, 200 μL of above culture suspension was added. The tubes were incubated at 37 °C for 24 h for bacteria using NB as a control. In order to ensure that the solvent had no effect on growth, control without test samples and with solvent (DMSO) was assayed simultaneously. All the tubes were examined for their visible turbidity. The lowest concentration was noted as MIC at which no visible growth was observed.

## Declarations

### Author contribution statement

Soukhyarani G. Nayak: Conceived and designed the experiments; Performed the experiments; Analyzed and interpreted the data; Contributed reagents, materials, analysis tools or data; Wrote the paper.

Boja Poojary: Analyzed and interpreted the data; Contributed reagents, materials, analysis tools or data.

### Funding statement

This research did not receive any specific grant from funding agencies in the public, commercial, or not-for-profit sectors.

### Competing interest statement

The authors declare no conflict of interest.

### Additional information

No additional information is available for this paper.
